# Functional Status, Meanings of Life Activities, and COVID-19 Related Disruptions among Disabled Older Adults

**DOI:** 10.1093/geroni/igaa057.3469

**Published:** 2020-12-16

**Authors:** Meng-Hsuan Yu, Shiau-Fang Chao

**Affiliations:** 1 National Taiwan University, Taipei City, Taipei, Taiwan (Republic of China); 2 National Taiwan University, Taipei City, Taiwan (Republic of China)

## Abstract

Participating in meaningful activities has been proven beneficial to the well-being of disabled older adults. However, social distancing policies and restrictions on public activities have been implemented since the outbreak of COVID-19 at the beginning of 2020 in Taiwan. These restrictions not only prevent older individuals from performing meaningful activities but also have actual impacts on their daily life. This study aims to elucidate the intervening role of meanings of life activities on the relationship between functional status and COVID-19 disruptions. Data were collected from a sample of 526 community-dwelling older adults with disabilities in Taiwan between April and July, 2020. Utilizing Multiple Regression Analysis, the research findings were as follows. First, participants with better functional status experienced more COVID-19 related disruptions to their daily routine. In the meantime, they also valued their life activities as more meaningful than those with worse functional status. Second, higher levels of meanings in performing life activities also positively related to COVID-19 disruptions. Third, meanings of life activities fully mediated the relationship between functional status and COVID-19 disruptions. That is, disabled older individuals with better functional status may experience more COVID-19 related disruptions because their accessibility to meaningful activities was limited. Since preventive approaches to control the spread are necessary during COVID-19 epidemic, efforts should be made to sustain meaningful life activities participation among disabled older adults. Based on the findings of this study, this would be especially critical to the well-being of more capable older individuals with disabilities during the pandemic.

